# Relationship of Socio Economic Status, Income, and Education with the Survival Rate of Breast Cancer: A Meta-Analysis

**Published:** 2019-08

**Authors:** Majid TAHERI, Mohammad TAVAKOL, Mohammad Esmaeil AKBARI, Amir ALMASI-HASHIANI, Mahmoud ABBASI

**Affiliations:** 1. Medical Ethics and Law Research Center, Shahid Beheshti University of Medical Sciences, Tehran, Iran; 2. Department of Sociology, School of Social Sciences, University of Tehran, Tehran, Iran; 3. Cancer Research Center (CRC), Shahid Beheshti University of Medical Sciences, Tehran, Iran; 4. Department of Epidemiology, School of Health, Arak University of Medical Sciences, Arak, Iran; 5. Traditional and Complementary Medicine Research Center, Arak University of Medical Sciences, Arak, Iran

**Keywords:** Breast cancer, Social determinant of health, Meta-analysis, Review

## Abstract

**Background:**

Despite our awareness of the significant effect of Social Determinant of Health (SDoH) such as Socio Economic Status (SES), income and education on breast cancer survival, there was a serious lack of information about the effect of different level of these factors on breast cancer survival. So far, no meta-analysis has been conducted with this aim, but this gap was addressed by this meta-analysis.

**Methods:**

Main electronic databases such as PubMed, Web of Science, and Scopus were investigated up to January 2019. Epidemiological studies focusing on the association between SDoH and breast cancer were singled out. Q-test and I^2^ statistic were used to study the heterogeneity across studies. Begg’s and Egger’s tests were applied to explore the likelihood of the publication bias. The results were reported as hazard ratio (HR) with 95% confidence intervals (CI) through a random-effects model.

**Results:**

We identified 7,653 references and included 25 studies involving 1,497,881 participants. The HR estimate of breast cancer survival was 0.82 (0.67, 0.98) among high level of SES, 0.82 (0.70, 0.94) among high level of income and 0.72 (0.66, 0.78) among academic level of education.

**Conclusion:**

The SES, income, and education were associated with breast cancer survival, although the association was not very strong. However, there was a significant association between the levels of these factors and breast cancer survival.

## Introduction

Breast cancer is the most common cancer in developed and developing countries (1). It is the second most common cause of death due to cancer after lung cancer in women (2). According to recent statistics, the incidence of this cancer is increasing globally about 2% per year (3). Increasing of BC survival rate were showed in some studies (4–6). Improved survival rate is probably attributed to progression of treatment and increased screening (7). However, unfortunately, not all women enjoy such an increase in survival rate. This problem can be due to individual differences. Results of previous studies showed that the SDoH had an effect on survival, morbidity, and mortality rate of cancers (8). The diagnostic and therapeutic methods for disease have improved. However, the morbidity, mortality and prevalence of disease has been affected by the SDoH (8). Some of these factors include childhood conditions, social status, addiction, social support, work environment, transportation, etc. (9). Some indices are used for assessing these factors such as the socioeconomic status, level of education, occupation and level of income (10). The survival rate of breast cancer in countries with low level of education is often lower than other countries. The death induced by breast cancer in patients with low levels of education is 1.39 times higher in patients with high levels of education (11). Diagnosis, treatment and prognosis of breast cancer patients with low socioeconomic status were lower and poorer than other (12).

Despite our awareness of the effect of some items of SDoH on breast cancer survival, data on effect of different level of these factors on breast cancer survival is scarce. This gap was addressed by this meta-analysis. To date, several studies have been performed on the survival rate of breast cancer and the effect of SDoH on it (13–16). Despite the existence of these primary studies, no meta-analysis has been performed yet to investigate the effect of SDoH on breast cancer survival rate. This meta-analysis conducted to explore the prospective cohort studies, carried out in diverse settings, to assess the extent of effect of some SDoH factors on breast cancer survival rate.

## Materials and Methods

This meta-analysis was approved and funded by the Vice-Chancellor of Research and Technology, Shahid Beheshti University of Medical Sciences.

### Inclusion criteria

Cohort studies focusing on the survival rate of patients suffering from breast cancer were put into this meta-analysis, irrespective of publication date, language, age, nationality, religion, and race. The search was not in terms of language, but all the selected appropriate references in this meta-analysis were in English. One outcome was considered: the effect of some items of SDoH (included: education level, income and Socio Economic Status (SES)) on survival rate of breast cancer.

A case of breast cancer was specified with kind of tissue cancer that chiefly deals with the inner layer of milk glands or lobules, and ducts (tiny tubes that carry the milk) (17). As specified with WHO, The SDoH cover the major features of life and job like SES, employment, insurance status, education, and race influencing one’s health both directly and indirectly (18).

### Search methods

The following keyword set was used to develop the search strategy: (survival or survive or mortality or death) and (breast cancer or breast malignancy or breast tumor or breast neoplasm) and (cohort, retrospective, prospective, or follow-up or longitude) and (education level or income or employment or job or economic stability or food security or SDoH). Electronic databases including PubMed, Web of Science, and Scopus were searched until January 2019. To find extra references, the reference lists of all included studies were explored. Additionally, the authors of the selected studies were called for extra-unpublished studies.

### Data collection and analysis

Two authors (MT and AAH) made the independent decision as to which studies should be put into this meta-analysis according to the study inclusion criteria. The probable disagreements were handled by discussion between the authors until reaching consensus. The kappa statistic for between-author reliability was 83%. Two authors (MT and AAH) extracted the data from the included studies. Once more, the probable disagreements were handled by discussion between the authors until reaching consensus. The variables extracted for analysis included first author’s name, year and country of study, mean (range) age, study design, controlling for confounding (adjusted, unadjusted), sample size, items of SDoH and effect size associated 95% of confidence interval (CI).

### Methodological quality

Newcastle Ottawa Statement (NOS) Manual (19) was applied to examine the included studies in terms of the reporting quality. The NOS scale has a checklist of items to determine the risk of bias in the included studies and assigns a maximum of nine stars to the following domains: selection, comparability, exposure, and outcome. In this meta-analysis, the studies having seven star items or more were high-quality studies and those having six star items or less were low-quality ones.

### Heterogeneity and publication bias

Statistical heterogeneity was explored using chi-squared test at the 95% of significance level (P < 0.05). Inconsistency between the results of studies was quantified using I^2^ (20). The Begg’s (21) and Egger’s (22) tests were used to check the likely publication bias. The survival rate (P) and its related 95% of confidence interval (CI) were shown as measures of survival rate from breast cancer influenced by each items of specified SDoH through a random-effects model (23). All statistical analyses were carried out at a significance level of 0.05 by Stata software (Version, 11, StataCorp, College Station, TX, USA).

### Summary measures

We reported the relationship between some items of SDoH and breast cancer survival using hazard ratio (HR) with their 95% of confidence intervals (CI). Wherever reported, we applied the adapted form of HR measured for three potential confusing factors included age, race, and tumor size.

## Results

### Description of studies

**Fig. 1:**
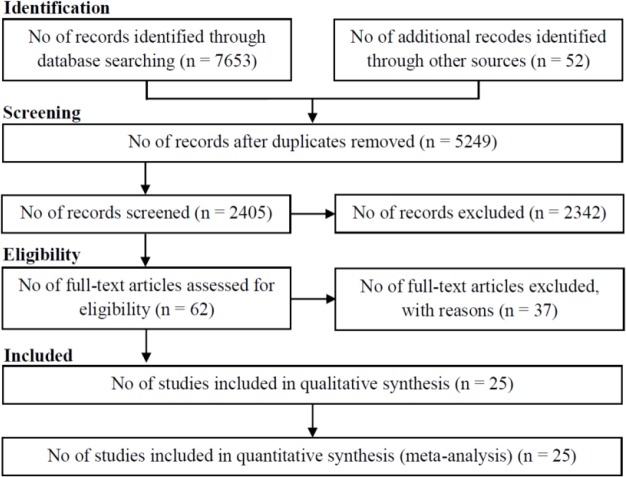
Flow of information through the different phases of the systematic review

The search singled out 7653 studies involving 7602 references from electronic databases, and 52 references from reference lists. Overall, 5249 duplicates, 2342 references were unrelated by reading titles and abstracts, and 37 references not fulfilling the inclusion criteria were left out. Thus, 25 studies, including 1,497,881 participants, were put into this meta-analysis (Fig. 1). The features of the selected studies are shown in Table 1. Of the 25 selected studies in this meta-analysis, all had a cohort design; 22 were prospective (13–16, 24–41) and 3 retrospective (42–44).

**Table 1:** Summary of studied articles

***First author (yr)***	***Country***	***Design***	***Mean age (years)***	***Effect size***	***Sample size***	***NOS quality***
Agarwal, Sh 2017	USA	Prospective	69.7	Hazard Ratio	362797	*******
Angeles, A 2016	Mexic	Retrospective	69.3	Hazard Ratio	854	********
Arias-Oritz, N 2018	Colombia	Prospective	63.4	Hazard Ratio	375	******
Brooke, H 2017	Sweden	Prospective	72	Hazard Ratio	14231	*******
Chang, Ch 2012	Taiwan	Prospective	57.6	Hazard Ratio	3223	*****
Dabbikeh, A 2017	Canada	Retrospective	59.3	Hazard Ratio	920334	*******
Dalton, S 2007	Denmark	Prospective	76.5	Hazard Ratio	25897	*******
Davoudi, E 2017	Iran	Prospective	62.5	Hazard Ratio	797	********
Diniz, R 2016	Brazil	Prospective	71.3	Hazard Ratio	459	*******
Du, X 2008	USA	Prospective	74.9	Hazard Ratio	35029	*****
Eaker, S 2009	Sweden	Prospective	71.5	Hazard Ratio	9908	******
Feller, A 2017	Swiss	Prospective	57.5	Hazard Ratio	10915	*******
Gajalakshmi, CK 1997	India	Prospective	76.9	Hazard Ratio	2080	*******
Goldberg, M 2015	Israeel	Prospective	60.2	Hazard Ratio	21034	********
Hastert, T 2015	USA	Prospective	58.5	Hazard Ratio	25260	******
Hussain, Sh 2008	Sweden	Prospective	68.4	Hazard Ratio	5718	*****
Lagerlund, M 2005	Sweden	Prospective	79.1	Hazard Ratio	8230	******
Lan, N 2013	Vietnam	Retrospective	64.5	Hazard Ratio	799	*******
Larsen, S 2015	Danmark	Prospective	63.5	Hazard Ratio	1229	*******
Miki, Y 2014	Japan	Prospective	62.7	Hazard Ratio	22458	********
Rezaianzadeh, A 2009	Iran	Prospective	69.9	Hazard Ratio	1148	*****
Schrijvers, C 1995	Netherland	Prospective	68.8	Hazard Ratio	3928	******
Shariff, S 2015	USA	Prospective	60.8	Hazard Ratio	9372	*******
Stavraky, K 1996	Canada	Prospective	54.9	Hazard Ratio	575	********
Teng, A 2017	New Zealand	Prospective	59.3	Hazard Ratio	11231	*******

### Results of the search

In this study, 10,687 references were recognized involving 10,159 references by the electronic searches and 528 by screening reference lists or calling the target authors up to July 2015. Overall, 3590 duplicates were left out and 7003 obvious unrelated references by reading titles and abstracts. Hence, 94 references remained for further assessment. Sixty references were left out because of not fulfilling the inclusion criteria. Thirty-four studies fulfilled our inclusion criteria (Fig. 1). NOS manual was applied to explore the selected studies in terms of reporting quality. Based on this scale, 15 studies were high quality (13, 14, 16, 26–28, 31–33, 36, 39, 40, 42–44) and 10 studies were low quality (15, 24, 25, 29, 30, 34, 35, 37, 38, 41).

### Synthesis of results

**Fig. 2:**
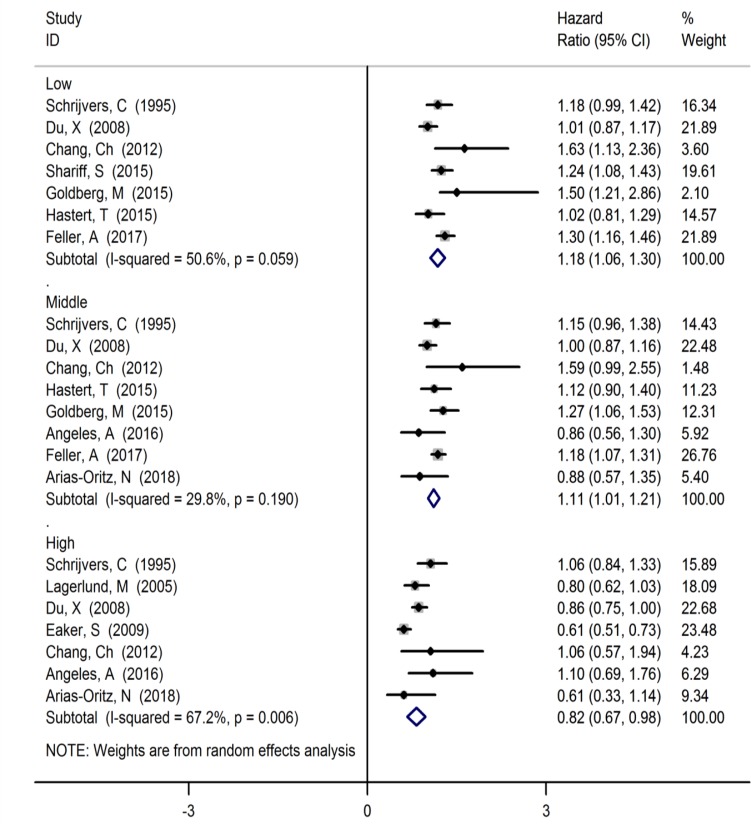
Forest plot of the relationship between of levels of SES with breast cancer survival

**Fig. 3:**
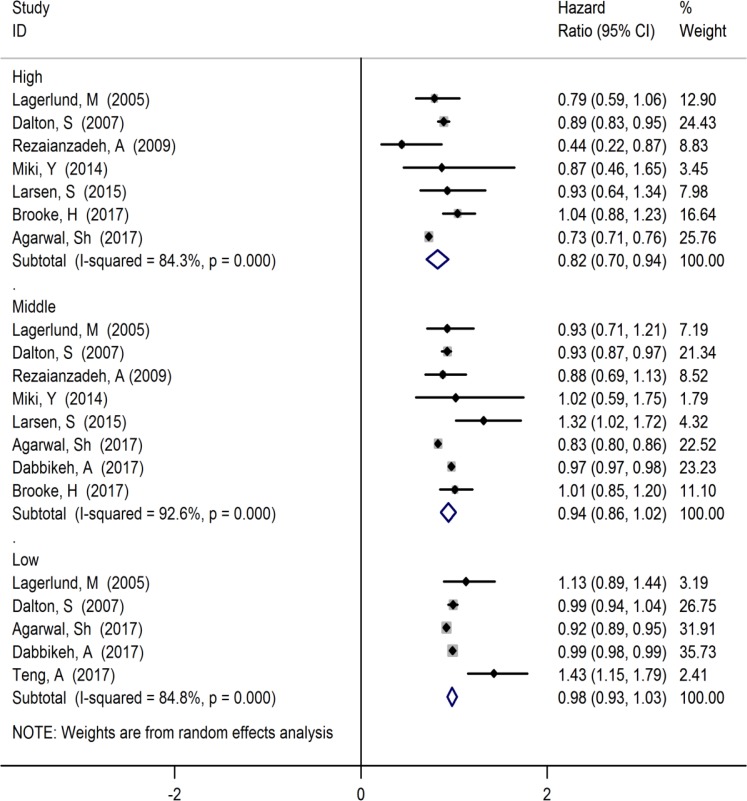
Forest plot of the relationship between of levels of income with breast cancer survival

**Fig. 4:**
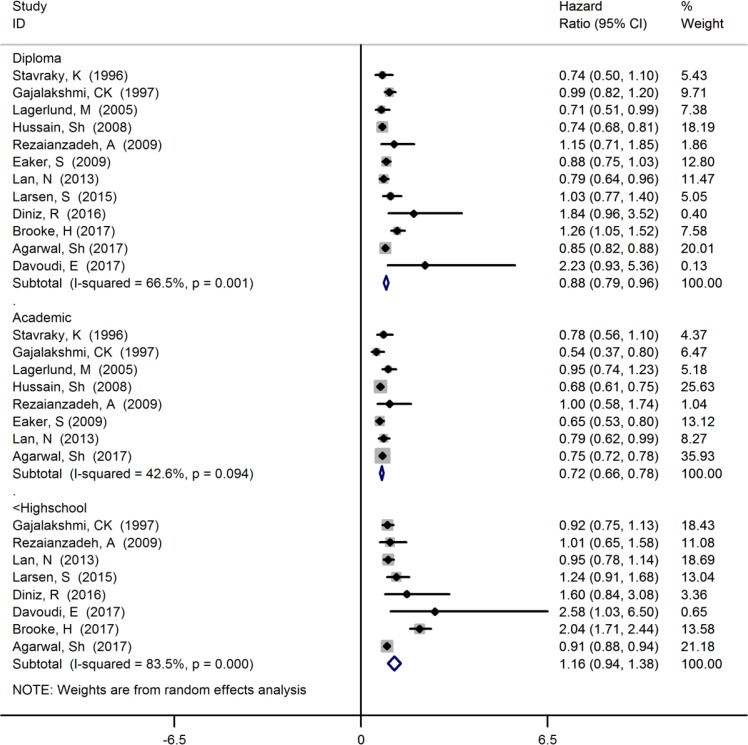
Forest plot of the relationship between of levels of education with breast cancer survival

The relationship between breast cancer survival and some items of SDoH is given in Figs. 2–4. Based on these forest plots, the HR estimate of breast cancer survival was 1.18 (95% CI: 1.06, 1.30, I^2^=50.6%, 7 studies) among low level of SES, 1.11 (95% CI: 1.01, 1.21, I^2^=29.8%, 9 studies) among middle level of SES and 0.82 (95% CI: 0.67, 0.98, I^2^=67.2%, 7 studies) among high level of SES (Fig. 2).

The HR estimate of breast cancer survival was 0.98 (95% CI: 0.93, 1.03, I^2^=84.8%, 5 studies) among low level of income, 0.94 (95% CI: 0.86, 1.02, I^2^=92.6%, 8 studies) among middle level of income and 0.82 (95% CI: 0.70, 0.94, I^2^=84.3%, 7 studies) among high level of income (Fig. 3).

The HR estimate of breast cancer survival was 1.16 (95% CI: 0.94, 1.38, I^2^=83.5%, 8 studies) among under the high school level of education, 0.88 (95% CI: 0.79, 0.96, I^2^=66.5%, 12 studies) among diploma level of education and 0.72 (95% CI: 0.66, 0.78, I^2^=42.6%, 8 studies) among academic level of education (Fig. 4).

### Publication bias

The Begg’s and Egger’s tests were used to examine the likely publication bias that was further visualized by the funnel plot. No indication of publication bias was observed according to the Begg’s test among studies focusing on the breast cancer survival among levels of SES (P=0.414), levels of income (P=0.516), and levels of education (P=0.161). Moreover, according to the results of Egger’s test, there was no evidence of publication bias among studies addressing the breast cancer survival among levels of SES (P=0.573), levels of income (P=0.158), and levels of education (P=0.183).

## Discussion

We summarized the available evidence from cohort studies addressing the relationship between some items of SDoH and breast cancer survival. Our results suggested that the high level of SES, income and education were considerably related with higher survival of breast cancer; however, this association was not very strong.

Evidence of heterogeneity was observed in all the included studies but for studies reporting the HR of breast cancer among low and middle levels of SES and academic level of education. The difference between the participants and the risk of bias of the included studies can be used to justify the observed heterogeneity relatively. However, care must be taken in the Q-test interpretation. One of problems of this test may occur in meta-analysis of observational studies with large sample size. The involvement of many studies in a meta-analysis, as in our meta-analysis, means more power of the test to spot a small amount and clinically unimportant of heterogeneity (45). The small within-study variance is one reason that may explain this heterogeneity. In addition, the issue that the studies come from different settings and different populations sample sizes and follow-up periods are the other reasons clarifying the observed heterogeneity across studies relatively.

In this study, an increase in the levels of education leads to an increase in the survival of breast cancer. The effect of levels of education on health status has been performed in previous studies (46). The results of a study expressed that the low education levels was a risk factors for death of breast cancer (47). The levels of education also are related to the levels of income and SES. In other words, the difference in educational levels reflects the difference in SES between individuals. These differences affect the health status of individuals in a variety of ways. Women with higher levels of education show up for screening for breast cancer regularly and frequently (48). As a result, their cancers will be diagnosed sooner. In the present study, the association between SES and BC survival was significant. This association also was significant in various studies. The dose-response relationship between SES and education levels with BC survival were showed in this study. The BC survival increased with the increasing the SES and education levels. The dose-response relationship is an important criterion of causality. In a study, the education levels had a positive effect on BC survival. In addition, the BC patients with higher SES had a more survival (49). Another study evaluated the SES effect on survival of breast cancer in young Australian urban women. It showed that the HR estimate of BC recurrence in women with academic education compared to incomplete high school education was 1.51; however, it was not significant. In addition, there was not association between SES and BC survival that was not consistent with our findings (50). In a study conducted by Aziz et al. the patients with BC were divided to three groups (low, middle, and high SES). The lower SES had an effect on BC survival. In addition, the association between education levels and BC survival was determined (51). There was SES inequality in BC survival rate (52). The diagnosis and screening of breast cancer related to SES levels and increased the BC survival.

Moreover, these individuals are often at higher levels of income and can receive the best health care services easily and quickly. The survival rate of breast cancer is associated with comprehensive treatment. In addition, individuals with higher levels of education study more; therefore, they have more information about screening, prevention, and treatment of cancer.

The lower use of mammography screening leads to higher late stage diagnosed cancers and lower the survival rate. The survival of cancer also is affected by age at diagnosis and tumor progression. In this study, the effect of the SES, income, and education levels on survival rate of breast cancer calculated with adjust for age and tumor progression.

There were some limitations in this study as follows: i) the primary objective of this meta-analysis was to evaluate the effect of all items of SDoH on breast cancer survival, but because of restricted number of studies for other items (employment, access to health center, economic stability and food security), finally we only included these three items (SES, education and income). ii) Other limitations of this study included inadequate prospective cohort study, low-quality studies, and unavailability of the studies for diverse reasons like outdated studies and not printed electronic papers. iii) We performed subgroup analysis to assess the effect of different level of SES, education and income (low, middle, high) on breast cancer survival. Nevertheless, the number of studies in some subgroups restricted. This probably influenced the reliability of the results of subgroup analyses. Regardless of these limitations, this meta-analysis could find evidences of association between breast cancer survival and SES, education and income.

In addition, this meta-analysis indicated an apparent relationship between the levels of SES, education and income with breast cancer survival. The amount of studies and body of identified evidence made a robust conclusion possible regarding the objective of the study for estimating the effect of these items of SDoH; seemingly, it is not probable that further research would change our confidence in the estimate of effect.

## Conclusion

The levels of SES, education and income were significantly associated with breast cancer survival, although these associations were not very strong. Nevertheless, this issue justifies that increased level of these factors may help increased the survival of cancers including breast cancer.

## Ethical considerations

Ethical issues (Including plagiarism, informed consent, misconduct, data fabrication and/or falsification, double publication and/or submission, redundancy, etc.) have been completely observed by the authors.

## References

[1] Bray F Ren JS Masuyer E Ferlay J (2013). Global estimates of cancer prevalence for 27 sites in the adult population in 2008. Int J Cancer, 132(5):1133–45. 22752881 10.1002/ijc.27711

[2] Zaorsky NG Churilla TM Egleston BL (2017). Causes of death among cancer patients. Ann Oncol, 28(2): 400–407. 27831506 10.1093/annonc/mdw604 PMC5834100

[3] DeSantis C Siegel R Bandi P Jemal A (2011). Breast cancer statistics, 2011. CA Cancer J Clin, 61(6): 409–418. 21969133 10.3322/caac.20134

[4] Diaz de la Noval B Frias Aldeguer L (2018). Increasing survival of metastatic breast cancer through locoregional surgery. Minerva Ginecol, 70(1): 44–52. 28994557 10.23736/S0026-4784.17.04097-7

[5] Guo F Kuo YF Shih YCT (2018). Trends in breast cancer mortality by stage at diagnosis among young women in the United States. Cancer, 124(17): 3500–3509. 30189117 10.1002/cncr.31638 PMC6191354

[6] Yoshimura A Ito H Nishino Y (2018). Recent Improvement in the Long-term Survival of Breast Cancer Patients by Age and Stage in Japan. J Epidemiol, 28(10): 420–427. 29479003 10.2188/jea.JE20170103 PMC6143379

[7] Berry DA Cronin KA Plevritis SK (2005). Effect of screening and adjuvant therapy on mortality from breast cancer. N Engl J Med, 353(17):1784–92. 16251534 10.1056/NEJMoa050518

[8] Link BG Phelan JC (1996). Understanding sociodemographic differences in health--the role of fundamental social causes. Am J Public Health, 86(4): 471–473. 8604773 10.2105/ajph.86.4.471 PMC1380543

[9] Maia MF Kliner M Richardson M Lengeler C Moore SJ (2018). Mosquito repellents for malaria prevention. Cochrane Database Syst Rev, 2:CD011595. 29405263 10.1002/14651858.CD011595.pub2 PMC5815492

[10] Kelly MP Bonnefoy J Morgan A Florenzano F (2006). The development of the evidence base about the social determinants of health. Geneva: World Health Organization.

[11] Bouchardy C Verkooijen HM Fioretta G (2006). Social class is an important and independent prognostic factor of breast cancer mortality. Int J Cancer, 119(5):1145–1151. 16557599 10.1002/ijc.21889

[12] Brooke HL Weitoft GR Talbäck M (2017). Adult children’s socioeconomic resources and mothers’ survival after a breast cancer diagnosis: A Swedish population-based cohort study. BMJ Open, 7(3): e014968. 10.1136/bmjopen-2016-014968 PMC5387936 28363931

[13] Agarwal S Ying J Boucher KM Agarwal JP (2017). The association between socioeconomic factors and breast cancer-specific survival varies by race. PLoS One, 12(12): e0187018. 29211739 10.1371/journal.pone.0187018 PMC5718412

[14] Hussain SK Altieri A Sundquist J Hemminki K (2008). Influence of education level on breast cancer risk and survival in Sweden between 1990 and 2004. Int J Cancer, 122(1): 165–169. 17708572 10.1002/ijc.23007

[15] Miki Y Inoue M Ikeda A (2014). Neighborhood Deprivation and Risk of Cancer Incidence, Mortality and Survival: Results from a Population-Based Cohort Study in Japan. PLoS One, 9(9): e106729. 25184297 10.1371/journal.pone.0106729 PMC4153661

[16] Ataollahi MR Sharifi J Paknahad MR Paknahad A (2015). Breast cancer and associated factors: a review. J Med Life, 8(Spec Iss 4):6–11. PMC5319297 28316699

[17] Braveman P Gottlieb L (2014). The social determinants of health: it’s time to consider the causes of the causes. Public Health Rep, 129 Suppl 2:19–31. 10.1177/00333549141291S206 PMC3863696 24385661

[18] Wells GA Shea B O’Connell D (2009). The Newcastle-Ottawa Scale (NOS) for assessing the quality of nonrandomised studies in meta-analyses. Ottawa Hospital Research Institute, Ontario.

[19] Higgins JPT Green S (2008). Cochrane handbook for systematic reviews of interventions Version 5.0.0 [updated February 2008]. The Cochrane Collaboration.

[20] Begg CB Mazumdar M (1994). Operating characteristics of a rank correlation test for publication bias. Biometrics, 50(4):1088–101. 7786990

[21] Egger M Davey SG Schneider M Minder C (1997). Bias in meta-analysis detected by a simple, graphical test. BMJ, 315(7109):629–634. 9310563 10.1136/bmj.315.7109.629 PMC2127453

[22] DerSimonian R Laird N (1986). Meta-analysis in clinical trials. Control Clin Trials, 7(3): 177–188. 3802833 10.1016/0197-2456(86)90046-2

[23] Arias-Ortiz NE de Vries E (2018). Health inequities and cancer survival in Manizales, Colombia: a population-based study. Colomb Med (Cali), 49(1): 63–72. 29983465 10.25100/cm.v49i1.3629 PMC6018827

[24] Chang CM Su YC Lai NS (2012). The combined effect of individual and neighborhood socioeconomic status on cancer survival rates. PLoS One, 7(8): e44325. 22957007 10.1371/journal.pone.0044325 PMC3431308

[25] Dalton SO Ross L Düring M (2007). Influence of socioeconomic factors on survival after breast cancer - A nationwide cohort study of women diagnosed with breast cancer in Denmark 1983–1999. Int J Cancer, 121(11):2524–2531. 17680561 10.1002/ijc.22979

[26] Davoudi Monfared E Mohseny M Amanpour F (2017). Relationship of Social Determinants of Health with the Three-year Survival Rate of Breast Cancer. Asian Pac J Cancer Prev, 18(4): 1121–1126. 28547951 10.22034/APJCP.2017.18.4.1121 PMC5494225

[27] Diniz RW Guerra MR Cintra JRD (2016). Disease-free survival in patients with nonmetastatic breast cancer. Rev Assoc Med Bras, 62(5): 407–413. 27656849 10.1590/1806-9282.62.05.407

[28] Du XL Fang SY Meyer TE (2008). Impact of treatment and socioeconomic status on racial disparities in survival among older women with breast cancer. Am J Clin Oncol, 31(2):125–132. 18391595 10.1097/COC.0b013e3181587890

[29] Eaker S Halmina M Bellocco R (2009). Social differences in breast cancer survival in relation to patient management within a National Health Care System (Sweden). Int J Cancer, 124(1): 180–187. 18844231 10.1002/ijc.23875

[30] Feller A Schmidlin K Bordoni A (2017). Socioeconomic and demographic disparities in breast cancer stage at presentation and survival: A Swiss population-based study. Int J Cancer, 141(8): 1529–1539. 28657175 10.1002/ijc.30856

[31] Gajalakshmi CK Shanta V Swaminathan R (1997). A population-based survival study on female breast cancer in Madras, India. Br J Cancer, 75(5): 771–5. 9043040 10.1038/bjc.1997.137 PMC2063339

[32] Goldberg M Calderon-Margalit R Paltiel O (2015). Socioeconomic disparities in breast cancer incidence and survival among parous women: findings from a population-based cohort, 1964–2008. BMC Cancer, 15:921. 26585765 10.1186/s12885-015-1931-4 PMC4653946

[33] Hastert TA Beresford SAA Sheppard L White E (2015). Disparities in cancer incidence and mortality by area-level socioeconomic status: a multilevel analysis. J Epidemiol Community Health, 69(2):168–176. 25288143 10.1136/jech-2014-204417

[34] Lagerlund M Bellocco R Karlsson P Tejler G Lambe M (2005). Socio-economic factors and breast cancer survival - A population-based cohort study (Sweden). Cancer Causes Control, 16(4): 419–430. 15953984 10.1007/s10552-004-6255-7

[35] Larsen SB Kroman N Ibfelt EH (2015). Influence of metabolic indicators, smoking, alcohol and socioeconomic position on mortality after breast cancer. Acta Oncol, 54(5):780–788. 25761087 10.3109/0284186X.2014.998774

[36] Rezaianzadeh A Peacock J Reidpath D (2009). Survival analysis of 1148 women diagnosed with breast cancer in Southern Iran. BMC Cancer, 9: 168. 19497131 10.1186/1471-2407-9-168 PMC2699348

[37] Schrijvers CTM Coebergh JW Van Der Heijden LH Mackenbach JP (1995). Socioeconomic variation in cancer survival in the Southeastern Netherlands, 1980–1989. Cancer, 75(12): 2946–2953. 7773946 10.1002/1097-0142(19950615)75:12<2946::aid-cncr2820751223>3.0.co;2-6

[38] Shariff-Marco S Yang J John EM (2015). Intersection of Race/Ethnicity and Socioeconomic Status in Mortality After Breast Cancer. J Community Health, 40(6): 1287–1299. 26072260 10.1007/s10900-015-0052-y PMC4628564

[39] Stavraky KM Skillings JR Stitt LW Gwadry-Sridhar F (1996). The effect of socioeconomic status on the long-term outcome of cancer. J Clin Epidemiol, 49(10):1155–1160. 8826996 10.1016/0895-4356(96)00179-5

[40] Teng AM Atkinson J Disney G (2017). Changing socioeconomic inequalities in cancer incidence and mortality: Cohort study with 54 million person-years follow-up 1981–2011. Int J Cancer, 140(6): 1306–1316. 27925183 10.1002/ijc.30555

[41] Angeles-Llerenas A Torres-Mejia G Lazcano-Ponce E (2016). Effect of care-delivery delays on the survival of Mexican women with breast cancer. Salud Publica Mex, 58(2): 237–250. 27557382 10.21149/spm.v58i2.7793

[42] Dabbikeh A Peng Y Mackillop WJ (2017). Temporal trends in the association between socioeconomic status and cancer survival in Ontario: a population-based retrospective study. CMAJ Open, 5(3):E682–E689. 10.9778/cmajo.20170025 PMC5621958 28877916

[43] Lan NH Laohasiriwong W Stewart JF (2013). Survival probability and prognostic factors for breast cancer patients in Vietnam. Glob Health Action, 6:1–9. 10.3402/gha.v6i0.18860 PMC3549066 23336619

[44] Higgins JPT Green S (2011). Cochrane handbook for systematic reviews of interventions Version 5.1.0 [updated March 2011]. The Cochrane Collaboration.

[45] Steenland K Henley J Thun M (2002). All-cause and cause-specific death rates by educational status for two million people in two American Cancer Society cohorts, 1959–1996. Am J Epidemiol, 156(1):11–21. 12076884 10.1093/aje/kwf001

[46] Herndon JE Kornblith AB Holland JC Paskett ED (2013). Effect of socioeconomic status as measured by education level on survival in breast cancer clinical trials. Psychooncology, 22(2): 315–323. 22021121 10.1002/pon.2094 PMC3920288

[47] Damiani G Basso D Acampora A (2015). The impact of level of education on adherence to breast and cervical cancer screening: Evidence from a systematic review and meta-analysis. Prev Med, 81:281–9. 26408405 10.1016/j.ypmed.2015.09.011

[48] Sprague BL Trentham-Dietz A Gangnon RE (2011). Socioeconomic status and survival after an invasive breast cancer diagnosis. Cancer, 117(7):1542–51. 21425155 10.1002/cncr.25589 PMC3116955

[49] Morley KI Milne RL Giles GG (2010). Socio-economic status and survival from breast cancer for young, Australian, urban women. Aust N Z J Public Health, 34(2):200–5. 23331366 10.1111/j.1753-6405.2010.00507.x PMC3556996

[50] Aziz Z Sana S Akram M Saeed A (2004). Socioeconomic status and breast cancer survival in Pakistani women. J Pak Med Assoc, 54(9): 448–53. 15518365

[51] Yu XQ (2009). Socioeconomic disparities in breast cancer survival: relation to stage at diagnosis, treatment and race. BMC Cancer, 9:364. 19828016 10.1186/1471-2407-9-364 PMC2770567

[52] Silber JH Rosenbaum PR Ross RN (2018). Disparities in Breast Cancer Survival by Socioeconomic Status Despite Medicare and Medicaid Insurance. Milbank Q, 96(4):706–754. 30537364 10.1111/1468-0009.12355 PMC6287075

